# Refining a Nordmøre-grid bycatch reduction device for the Spencer Gulf penaeid-trawl fishery

**DOI:** 10.1371/journal.pone.0207117

**Published:** 2018-11-21

**Authors:** Craig J. Noell, Matt K. Broadhurst, Steven J. Kennelly

**Affiliations:** 1 South Australian Research and Development Institute (Aquatic Sciences), West Beach, South Australia, Australia; 2 New South Wales Department of Primary Industries, Fisheries Conservation Technology Unit, Coffs Harbour, New South Wales, Australia; 3 IC Independent Consulting, Cronulla, New South Wales, Australia; Universidade Federal do Parana, BRAZIL

## Abstract

Incremental refinements were made to a generic Nordmøre-grid to minimise bycatches of blue swimmer crabs *Portunus armatus* and giant cuttlefish *Sepia apama*, while maintaining catches of western king prawns *Melicertus latisulcatus* in the Spencer Gulf penaeid-trawl fishery. These refinements involved varying bar spaces, escape-exit areas and guiding-panel lengths, and were compared against a conventional trawl. Catches of teleosts and *M*. *latisulcatus* largely remained unaffected by the changes. Maximum reductions in *P*. *armatus* and *S*. *apama* bycatches (both ~90%) were achieved with a Nordmøre-grid comprising 38-mm bar spaces, 0.81- or 1.05-m^2^ escape exits and a 2.7-m guiding panel. Catching fewer *P*. *armatus* should reduce abrasion and crushing of *M*. *latisulcatus* in the codend and so increase the value of this targeted species. While noting some unresolved operational concerns, these results demonstrate the potential improvements in selectivity in this fishery using a Nordmøre-grid, primarily by mechanical separation.

## Introduction

Relative to other fishing methods, penaeid trawling is generally considered to be poorly selective and can result in the discarding of large quantities of bycatch, which sometimes includes threatened or endangered species [1–3]. The most common technique for mitigating penaeid-trawl bycatch is to install physical modifications in the posterior trawl, termed bycatch-reduction devices (BRDs), which are designed to exclude organisms mainly based on differences in behaviour (‘behavioural separators’) or size (‘mechanical separators’) [4]. The latter category has been particularly successful in several fisheries, with designs like the ‘Nordmøre-grid’ consistently excluding up to 90% of total bycatch while maintaining catches of penaeids [5–8].

As for many penaeid-trawl fisheries over the past 20 years, those in Australia have made progressive attempts to refine mechanical separators to reduce the bycatch of various species of concern, including elasmobranchs and turtles [5, 9]. Currently, many Australian penaeid-trawl fisheries use mechanical separators and virtually all require behavioural separators [10–14]. One of the few remaining Australian penaeid-trawl fisheries that currently does not use any type of BRD occurs in Spencer Gulf, South Australia. The Spencer Gulf prawn fishery (SGPF) has a history of testing several mechanical-separating BRDs [15, 16], but while some significantly reduced bycatches, they did so inconsistently and unacceptable losses of the targeted western king prawn *Merlicertus latisulcatus* (mainly due to clogging of the grid with weed or crabs) precluded their implementation.

Currently, the fleet of the SGPF, which comprises 39 double-rigged trawlers (all using 2 × 14.6-m headline length trawls), typically trawl for ~50 nights per year [17] and in <20% of the gulf [18]. Fishers also are permitted to land southern calamari *Sepioteuthis australis* and slipper lobsters *Ibacus* spp., with these collectively known as ‘by-product’. All other incidentally caught species are discarded at sea. The SGPF has employed a range of strategies—both regulatory and voluntary—to manage the fishery, including bycatch avoidance, minimising discard mortality, fishing effort reduction (by ~60% over the past 40 years), spatial and temporal closures, and various on-board handling and discarding practices [19]. These strategies combined with other conservation efforts [20–24] have enabled the SGPF to gain accreditation by the Marine Stewardship Council (MSC) [25].

At some locations in Spencer Gulf, trawlers can encounter large densities of the blue swimmer crab *Portunus armatus* (averaging ~6.9–7.7 kg ha^-1^ trawled) [22, 23], whereas *P*. *armatus* catches are highly variable, sometimes exceeding catches of the targeted *M*. *latisulcatus*. The same stock of *P*. *armatus* is also exploited by commercial and recreational trap fisheries, with annual harvests in Spencer Gulf of ~380 and 290 t, respectively [26, 27]. In an attempt to mitigate *P*. *armatus* mortalities, all trawlers use a large-meshed ‘crab bag’ inside the codend, which effectively separates many *P*. *armatus* (and other large organisms) from *M*. *latisulcatus*, and thereby facilitates rapid discarding of the former, with assumed low mortality (~16%, based on estimates for another Australian fishery) [28, 29]. Notwithstanding few perceived impacts to discarded *P*. *armatus*, their exoskeleton and claws cause considerable damage to *M*. *latisulcatus* as the latter have to pass through the crab bag (and *P*. *armatus* retained in it) before reaching the end of the codend, reducing catch quality and value [15, 16, 21]. Ideally very few (if any) *P*. *armatus* should enter the crab bag and codend.

Another species that trawlers in Spencer Gulf interact with is the giant cuttlefish *Sepia apama*. *Sepia apama* is considered iconic in Australia and has attracted considerable attention in recent years because their annual spawning aggregation in northern Spencer Gulf (between May and July and is the largest known *Sepia* aggregation in the world) declined to record low levels in 2013 [30]. While successive increases in annual biomass indicated a recovering population [31], several studies were instigated at the time of the decline to investigate potential causes [32–34]. None of these provided any evidence that the SGPF had a detrimental impact on the *S*. *apama* population. Nevertheless, the fishing industry (i.e. licence holders) made a proactive decision to collaborate with researchers and managers to develop a Nordmøre-grid BRD to reduce bycatch of *S*. *apama* and *P*. *armatus* [7].

As a first step towards mitigating the bycatches of *P*. *armatus* and *S*. *apama* by the SGPF, Kennelly and Broadhurst [7] investigated the effective physical dimensions of a Nordmøre-grid for the trawls used. That study showed that a large (~1 × 2 m) low-angled (~30°) grid facilitated the escape of up to 34% (by number) of *P*. *armatus* and *Sepia* spp.—comprising *S*. *apama* and the morphologically similar, but smaller *S*. *novaehollandiae*—while maintaining catches of *M*. *latisulcatus*. Although promising, these individual species/group reductions were not as large as those observed for Nordmøre-grids tested and developed in other fisheries [6, 8, 35]—results that have been achieved through progressive design refinements of such grids.

The objective of this study was therefore to test incremental technical refinements to the Nordmøre-grid tested by Kennelly and Broadhurst [7] with regard to variables shown to affect the performance of similar designs elsewhere [8, 35]. Specifically, work was done to identify an optimal bar spacing for the grid (experiment 1) and then assess the utility of increasing the escape-exit area (experiments 2 and 3) and shortening the guiding panel (experiment 3). Performance of these refinements was assessed relative to three criteria of: (i) reducing total bycatch, with particular focus on maximising the escape of *P*. *armatus* and *S*. *apama*; (ii) maintaining and improving the quality of *M*. *latisulcatus* catches; and (iii) minimising technical handling issues.

## Materials and methods

**Fig 1:**
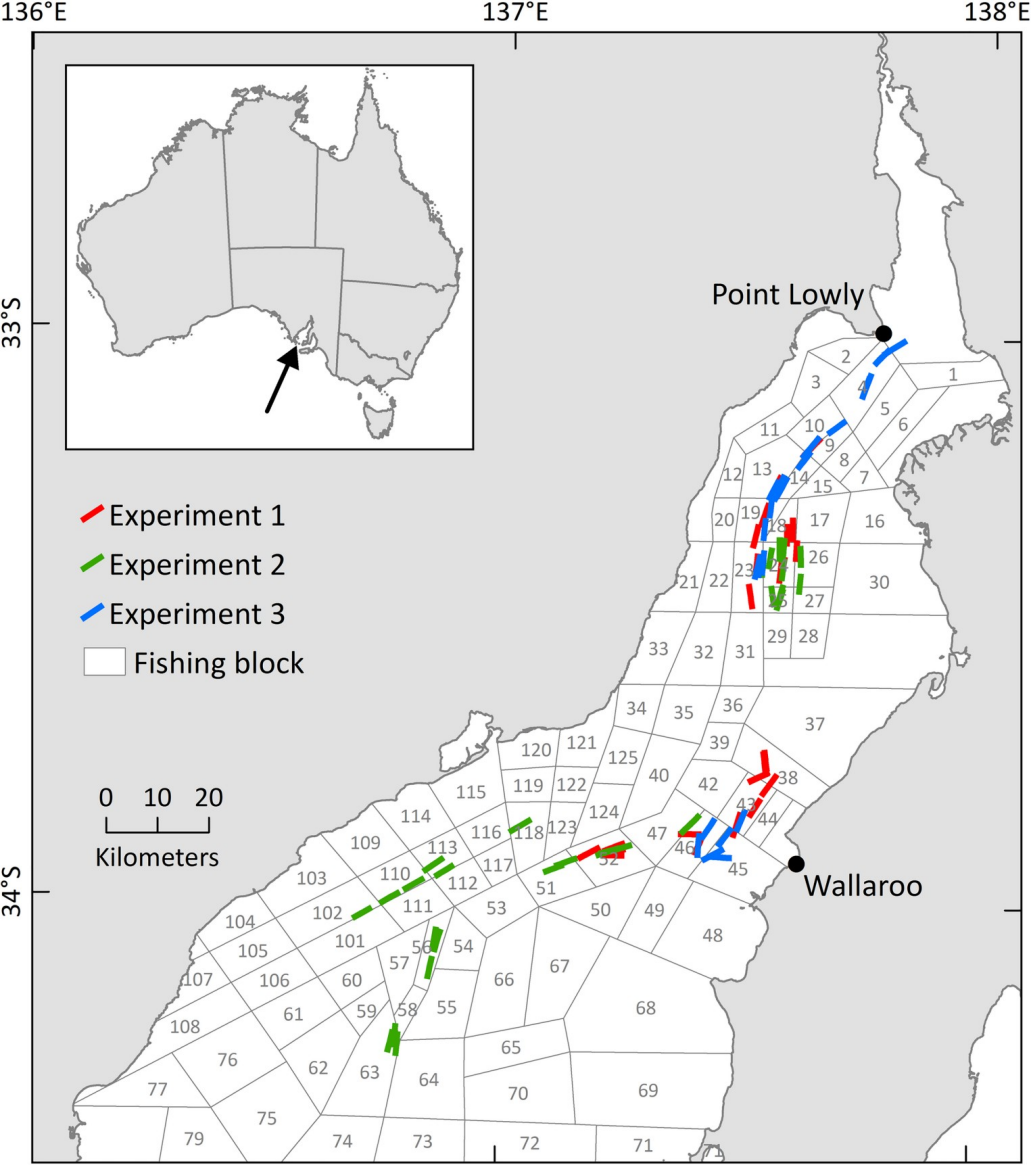
Map of northern Spencer Gulf showing the trawl paths of the study. Also shown are commercial fishing blocks (polygons). Inset map shows study location in Australia.

Three experiments were done during 13 nights (four in each of April 2015 and 2016, and five in November 2015) using the same double‐rigged trawler (22 m and 336 kW) in northern Spencer Gulf (32°59ʹ–34°17ʹS, 136°41ʹ–137°50ʹE; Fig 1) under ministerial exemption provided by Primary Industries and Regions South Australia (Exemption Nos 9902713 and 9902785). All experiments were done within traditional trawl grounds for *M*. *latisulcatus* in depths of 10–30 m across sandy substrata, specifically in regions where *P*. *armatus* and/or *S*. *apama* are known to occur (see S1 File for geographic coordinates of trawl paths). The trawler was rigged with two identical ‘Gundry’ trawl nets, each with a headline length of 14.63 m spread by flat‐rectangular otter boards (1.7 × 1.1 m) and towed at ~1.9 m s^‐1^. Both posterior trawl bodies were fitted with zippers (Buraschi S146R, 2.0 m long) to facilitate changing the extensions/codends described below.

### Nordmøre-grids and codends

**Fig 2:**
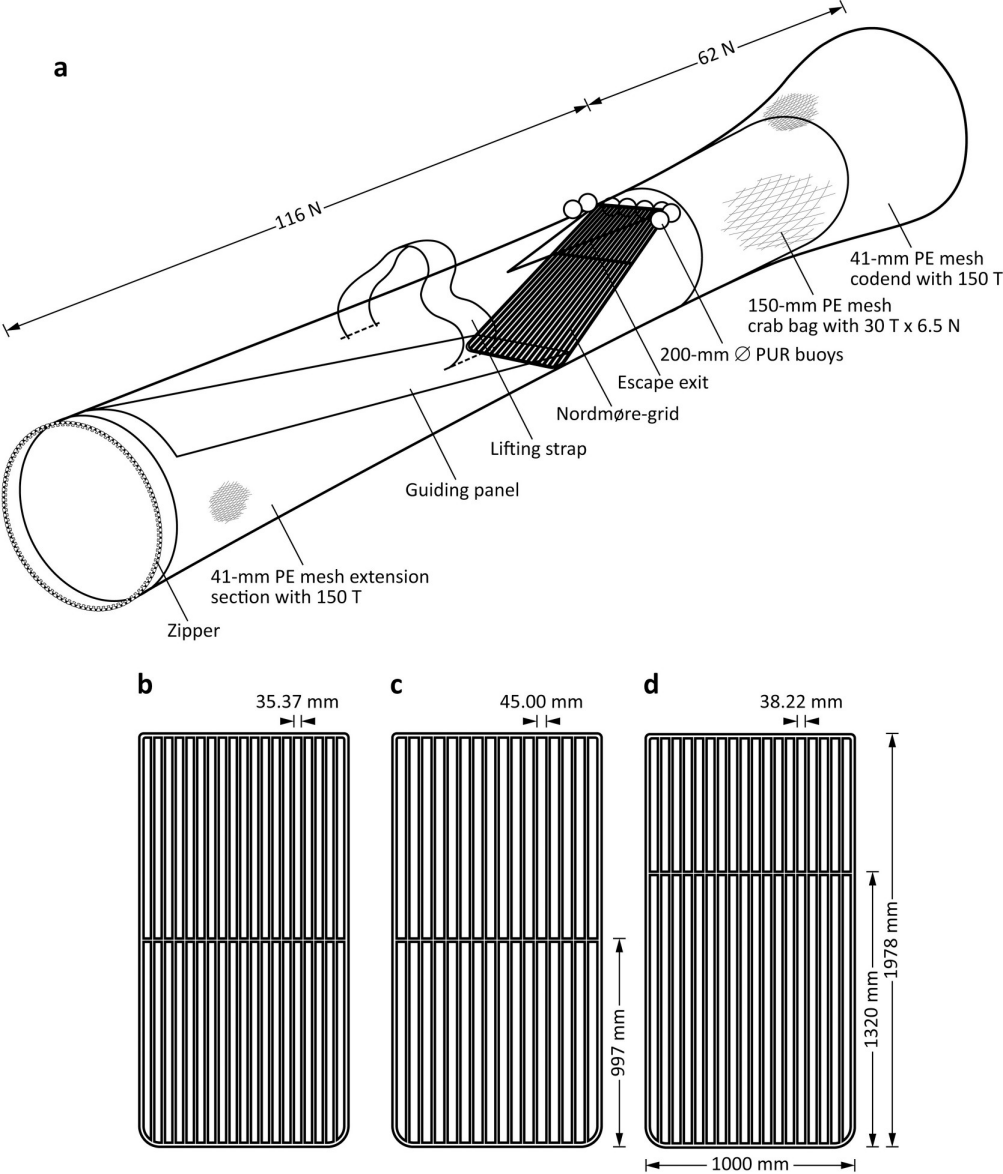
**Specifications of the (a) extension and codend (with crab bag) assembly with a Nordmøre‐grid installed and (b) 35-, (c) 45- and (d) 38-mm grids tested.** PE, polyethylene; PUR, polyurethane; T, transversals; N, normals; Ø, diameter.

One control (conventional) codend and six Nordmøre-grids and their extension sections/codends were constructed (Table 1; Fig 2). The control comprised 41‐mm (stretched mesh opening—SMO) mesh (2.2-mm diameter–Ø braided, green polyethylene–PE twine) measuring 105 meshes in the normal (N) direction and 150 meshes in the transverse (T) direction. Following conventional configurations, a cylindrical panel (termed a ‘crab bag’) of 150-mm SMO mesh (4.0-mm Ø braided green twine) and measuring 30 T × 6.5 N was inserted 32 N anterior to the last row of meshes in the codend to separate the larger *P*. *armatus* from *M*. *latisulcatus* (Fig 2A).

**Table 1:** Specifications of the Nordmøre‐grid treatments tested in each experiment.

Configuration	Experiment	Bar space (mm)	Horizontal support	Guiding panel length		Escape-exit	
				m	No. of meshes	Taper	Area (m^2^)
35-mm grid	1	35.37	Mid-point	3.3	75	AB	0.43
45-mm grid	1	45.00	Mid-point	3.3	75	AB	0.43
AB-exit 75N-panel grid	2	38.22	Top third	3.3	75	AB	0.43
1N2B-exit 75N-panel grid	2 and 3	38.22	Top third	3.3	75	1N2B	0.81
1N2B-exit 62N-panel grid	3	38.22	Top third	2.7	62	1N2B	0.81
1N1B-exit 62N-panel grid	3	38.22	Top third	2.7	62	1N1B	1.05

The 75- and 62-N guiding panels terminated at and 0.6 m anterior to the base of the grid, respectively.

All grids were flat, rectangular (1978 × 1000 mm) and constructed from solid aluminium rod (frame: 20‐mm Ø; bars: 16‐mm Ø) (Table 1; Fig 2B–2D). The key differences among grids were the spaces—dictated by the number of bars that could fit into the frame—and the location of a horizontal support (16-mm Ø rod) (Table 1; Fig 2B–2D). The first two grids (experiment 1) had 35.37- (termed the ‘35-mm grid’) and 45.00-mm (‘45-mm grid’) bar spaces and a centre horizontal rod (Table 1; Fig 2B and 2C). Based on their testing (see Results), the remaining four grids all had 38.22-mm bar spaces and their horizontal support located closer to the top (Table 1; Fig 2D).

**Fig 3:**
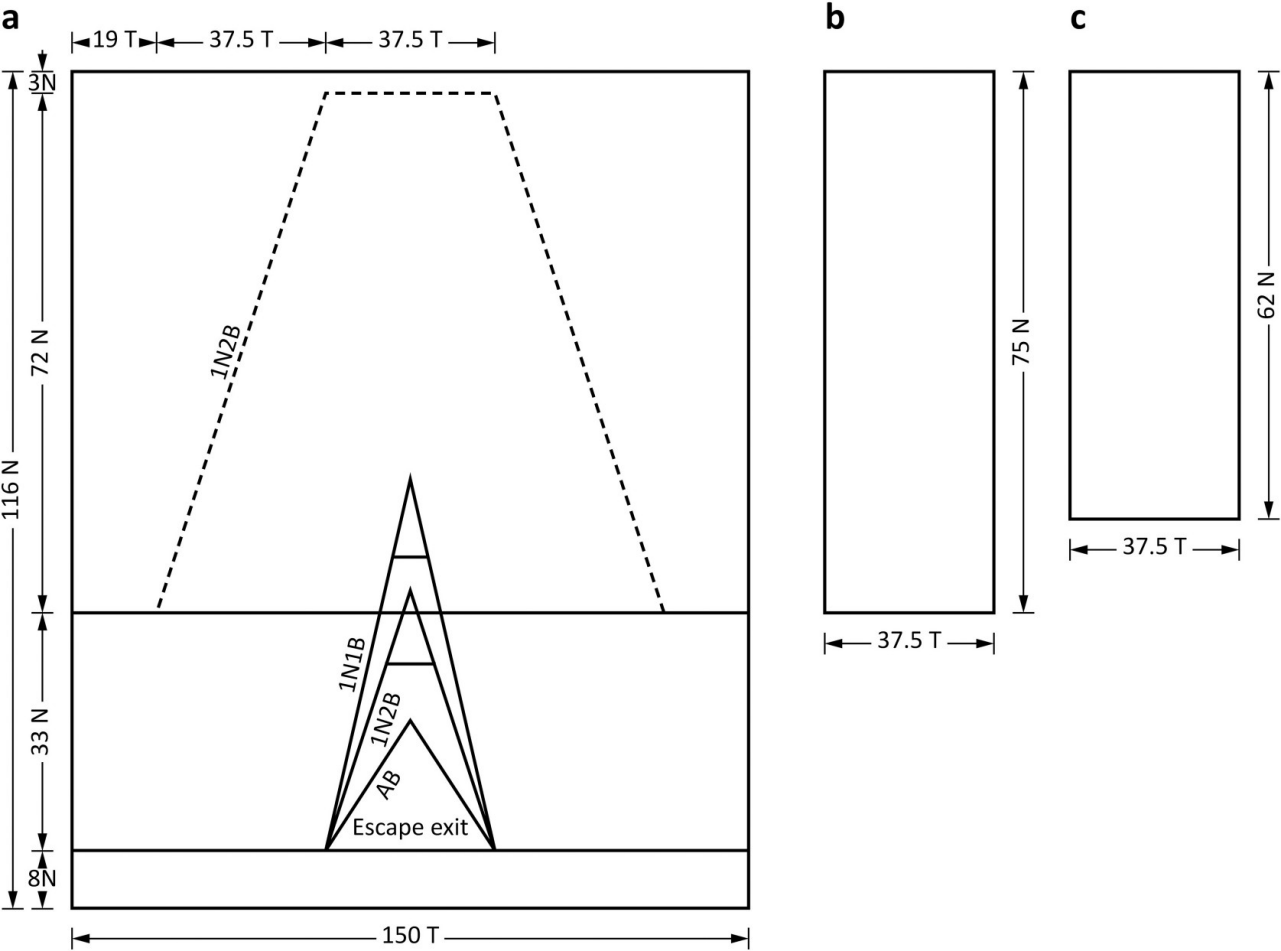
**Plans of the (a) extension cylinder (showing the AB-, 1N2B- and 1N1B-exit openings) and (b) 75-N and (c) 62-N guiding panels.** T, transversals; N, normal.

Each grid was inserted at ~30° into an extension section made from 41‐mm SMO mesh (3.0-mm Ø braided black PE twine) measuring 150 T × 116 N and with an anterior guiding panel (37.5 T wide; Table 1; Figs 2A and 3A–3C). The 35- and 45-mm grids both had 75-N (~3.3 m) guiding panels terminating at the grid base and triangular escape exits measuring 37 bars (B) on each side, providing an opening of 0.43 m^2^ (Table 1; Figs 2A and 3A and 3B).

The four 38-mm grids differed in their escape-exit areas and/or guiding-panel lengths. The first and second 38-mm grids had a 75-N guiding panel (as above); but while the first had a triangular escape exit (termed the ‘AB-exit 75N-panel grid’), the second had an escape exit made by cutting forward 1N2B either side, to provide an opening of 0.81 m^2^ (‘lN2B-exit 75N-panel grid’) (Table 1; Fig 3A). The third 38-mm grid also had a 1N2B escape exit, but with a shorter guiding panel (62 N and terminating 13 N or ~0.6 m anterior to the base of the grid; ‘1N2B-exit 62N-panel grid’), while the fourth 38-mm grid had a 1N1B escape exit (1.05 m^2^) and the shorter guiding panel (‘1N1B-exit 62N-panel grid’) (Table 1; Fig 3A and 3C).

Each Nordmøre-grid extension had lifting straps laterally attached 30 N anterior to the escape exit, sufficient 200-mm Ø polyurethane buoys (behind the top of the grid) to achieve neutral buoyancy, and a posterior codend (and crab bag) identical to the control, except it measured 62 N (Fig 2A). Zippers were attached to all grid extensions and the control codend to facilitate attachment and removal from the two trawl bodies (Fig 2A).

### Experimental design

The control was tested with the (i) 35- and 45-mm grids in experiment 1, (ii) AB- and 1N2B-exit 75N-panel grids in experiment 2, and (iii) 1N2B-exit 75N-panel and 1N2B- and 1N1B-exit 62N-panel grids in experiment 3 (Table 1). During each experiment, the control and grids being tested were alternately zippered to the nets on each side of the vessel and towed for 30 min. On each night, at least three replicates of each configuration were attempted through all possible combinations of pairings, although in experiment 2, some deployments were done where a treatment or control was not paired (i.e. another grid configuration was attached but was later omitted from analysis due to its non-identical codend).

### Data collected and statistical analyses

At the end of each deployment, the following data were collected from each codend: (i) total weights of *M*. *latisulcatus* by industry categories (i.e. sorted using a Haldane PTY grading machine), including ‘U8’, ‘U10’, ‘10–15’, ‘16–20’ and ‘21–30’ individuals per pound (454 g) and ‘soft and broken’ (i.e. post-moult and/or damaged *M*. *latisulcatus*); (ii) total weights of *S*. *australis* and Balmain bug *I*. *peronii*; (iii) total numbers, weights and, as an index of size in the absence of individual measurements, mean individual weights (total weight ÷ total number) [36] for *P*. *armatus*, *S*. *apama* and *S*. *novaehollandiae*; (iv) total weight of the remaining miscellaneous bycatch and its main components (elasmobranchs, porifera, algae/seagrasses and teleosts); and (v) numbers of individual teleost species. Representative subsampling was required to estimate the weights and numbers of *P*. *armatus* and teleosts in the miscellaneous bycatch, and to measure total lengths (TL, rounded down to the nearest 0.5 cm) of teleosts. In addition to the above quantitative data, at the end of each deployment any debris that collected in the extensions or at the escape exits was noted and cleared and information describing on-board handling of the grids was collected. In the event of an unsuccessful deployment, the likely technical reason was noted (where possible) and the deployment was repeated.

Catch variables within each experiment were separately assessed in linear mixed models (LMMs) with all log‐transformed, except the mean individual weights of key species, so that the predicted effects would be multiplicative. The LMMs included ‘configuration’ as a fixed effect, while ‘side’ of the vessel, ‘night’ and the interaction between ‘night’ and ‘deployment’ were included as random terms. Models were fitted using the ASReml-R package [37, 38] with the significance of configuration determined using a Wald *F*‐statistic. Significant effects of configuration were subsequently explored using the Benjamini‐Hochberg‐Yekutieli procedure to control the false-discovery rate (FDR) for multiple pairwise comparisons [39, 40]. Predicted means were back-transformed (where required from the log scale) to the original scale using the bias-correction formula of Sprugel [41].

To facilitate interpreting results between sequential trials, the hypothesis of no inter-experimental (*n* = 3) differences in the mean individual weights of *P*. *armatus*, *S*. *apama* and *S*. *novaehollandiae* was also tested. In these analyses, LMMs were fitted to data from the control only, with ‘experiment’ fixed, and night and side used as random terms. Significant differences were separated as above.

## Results

### Fishing conditions, catches and variability in mean sizes among experiments

Weather conditions during the three experiments (12–34 km h^-1^ winds and <1.3 m seas) and catches were typical of those experienced during conventional fishing. When pooled across experiments (147 replicate tows), the partitioned catches comprised 7152, 330 and 7377 kg of *M*. *latisulcatus*, by-product (*S*. *australis* and *I*. *peronii* combined) and bycatch, respectively (supplementary data, S1 Table). *Portunus armatus*, *S*. *apama* and *S*. *novaehollandiae* comprised 32.5, 2.1 and 0.9% of the total bycatch, respectively. While *P*. *armatus* and *S*. *novaehollandiae* maintained consistent trends between experiments, *S*. *apama* were more abundant during experiments 1 and 3.

**Fig 4:**
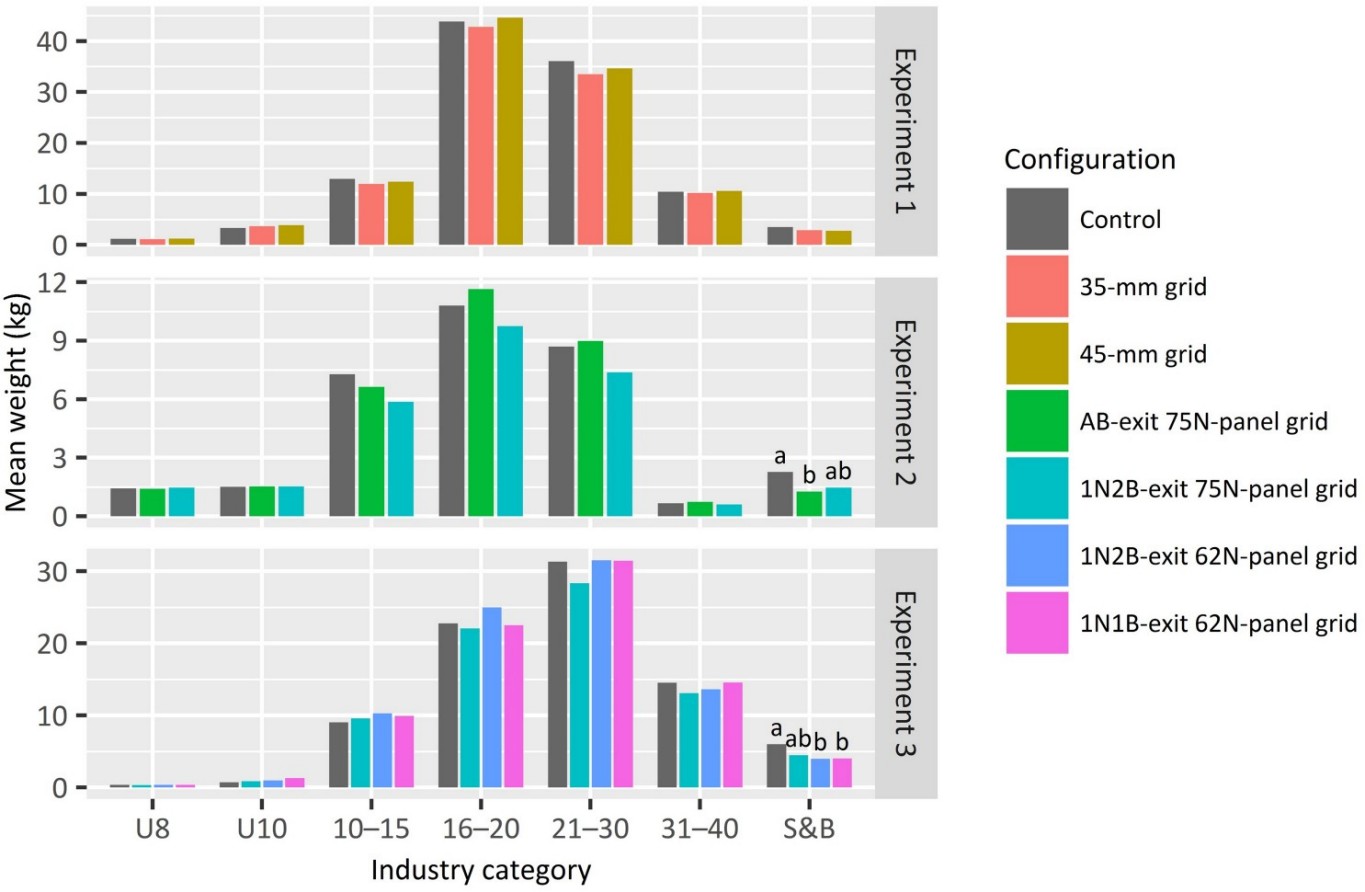
Predicted mean industry category weights 30 min^–1^ of *Melicertus latisulcatus* by configuration for experiments 1 (assessing bar spaces), 2 (assessing escape-exit area) and 3 (assessing escape area and guiding panel length). Where there was a significant effect (*p* < 0.05) among configurations within an experiment, letters above bars denote the false-discovery-rate (FDR) adjusted pairwise comparisons (*p* < 0.05).

Inter-experimental variability among the relative sizes of *M*. *latisulcatus* was minimal for the control (>60% of the catches had 16–30 individuals per pound; Fig 4). But the mean individual weight of *P*. *armatus* was significantly less in experiment 1 (100 ± 12 g) than experiment 2 (147 ± 12 g) (LMM and FDR, *p* < 0.05). By comparison, there were no significant differences in the mean individual weights of (i) *P*. *armatus* between experiments 2 (147 ± 12 g) and 3 (136 ± 13 g) (LMMs and FDRs, *p* > 0.05), (ii) *S*. *apama* between experiments 1 (432 ± 37 g) and 3 (489 ± 42 g) (too few were caught in experiment 2 –see below), or (iii) *S*. *novaehollandiae* among all three experiments (predicted means between 84 ± 15 and 102 ± 12 g).

The remaining bycatch in all three experiments comprised a similar suite and consistency of species—at least 48 teleosts, 11 chondrichthyans, 6 cephalopods, 6 crustaceans, 4 bivalves and 3 echinoderms (S1 Table). Of the teleosts caught in all experiments, most (90%) were small (<14 cm TL) monacanthids—predominantly rough leatherjacket *Scobinichthys granulatus* and bluefin leatherjacket *Thamnaconus degeni*, skipjack trevally *Pseudocaranx wrighti*, elongate bullseye *Parapriacanthus elongatus*, bluespotted goatfish *Upeneichthys vlamingii*, spotted dragonet *Repomucenus calcaratus* and silverbelly *Parequula melbournensis* (S1 Table).

### Experiment 1: Assessing bar spaces (control vs 35-mm vs 45-mm grids)

Despite the large size of the grids, few technical problems were encountered, with two deployments (one for each grid) repeated after the extension twisted during setting. However, debris was occasionally observed wedged between the top of the grid and the sides of the escape exit, which required cleaning. Where possible, this debris was retrieved on deck and included in catches of the main taxonomic groups. Although on-board handling of the grids was mostly straightforward, in one deployment a rock became dislodged and fell through the escape exit while the codend was being emptied.

**Fig 5:**
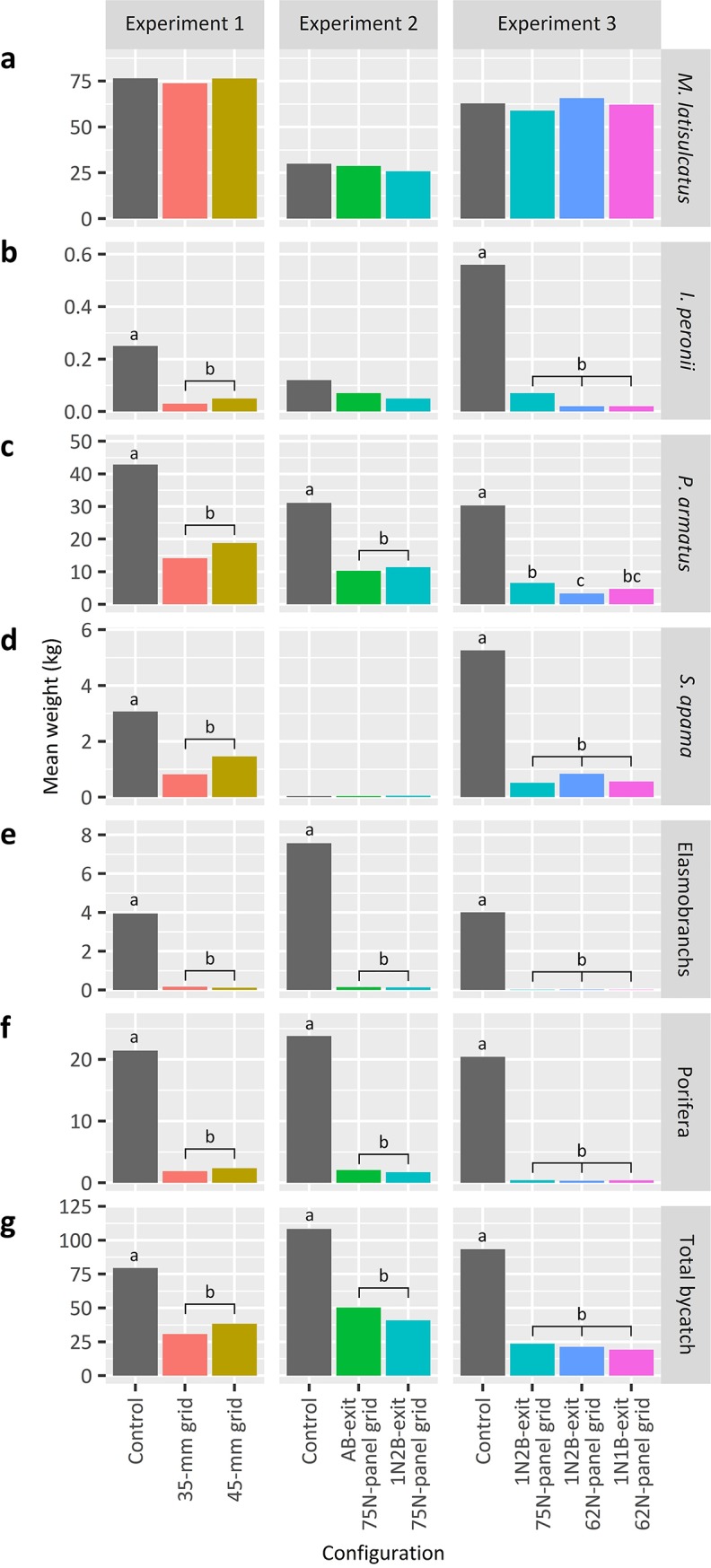
**Predicted mean catch weights 30 min**^**-1**^ **of (a) *Melicertus latisulcatus*, (b) *Ibacus peronii*, (c) *Portunus armatus*, (d) *Sepia apama*, (e) elasmobranchs, (f) porifera and (g) total bycatch by configuration and experiment.** Where there was a significant effect (*p* < 0.05) among a species/group within an experiment, letters above bars denote the false-discovery-rate (FDR) adjusted pairwise comparisons of the configurations (*p* < 0.05).

No significant differences were detected among configurations for any of the industry categories or total weights of *M*. *latisulcatus*, although slightly lower total catches were predicted with the 35-mm grid (by 4%) than either the 45-mm grid or control (LMM, *p* > 0.05; Table 2; Fig 5A). These results precipitated a decision to test the 38-mm bar space in subsequent experiments (Table 1). By comparison, significant configuration effects were detected for the numbers and weights of *P*. *armatus* and elasmobranchs, the number and weight of *S*. *apama*, and their mean individual weight, and weights of *I*. *peronii*, porifera and total bycatch (LMMs, *p* < 0.01; Table 2). No other variables (including all teleosts) were significantly affected by configuration (LMM, *p* > 0.05).

**Table 2:** Linear mixed model Wald *F*-values for the fixed effect of trawl configuration on log-transformed catches from experiments 1, 2 and 3.

Variable	Experiment 1	Experiment 2	Experiment 3
*Retained catches*			
Wt of *Melicertus latisulcatus*			
Total	0.61	2.73	1.03
U8^a^	0.20	0.04	0.77
U10^a^	0.74	0.00	3.15*
10–15^b^	0.93	2.25	0.71
16–20^b^	0.44	1.36	1.16
21–30^b^	0.52	3.69	1.05
31–40^b^	0.17	0.71	1.24
Soft and broken	2.76	5.14*	5.45**
Wt of *Sepioteuthis australis*	0.11	0.52	0.26
Wt of *Ibacus peronii*	11.64***	1.70	30.85***
*Discarded catches*			
Wt of total bycatch	40.61***	42.89***	48.73***
No. of elasmobranchs	18.78***	7.07**	95.28***
Wt of elasmobranchs	29.24***	60.86***	171.60***
Wt of porifera	28.40***	10.50***	37.47***
Wt of algae and seagrasses	0.34	2.07	1.29
Wt of teleosts	1.11	0.78	0.12
No. of *Portunus armatus*	16.14***	14.76***	17.59***
Wt of *P*. *armatus*	27.67***	15.74***	33.00***
Mean individual wt of *P*. *armatus*	3.14	4.00*	11.22***
No. of *Sepia apama*	5.86**	0.09	21.35***
Wt of *S*. *apama*	12.41***	0.20	20.08***
Mean individual wt of *S*. *apama*	8.14**	–	3.64*
No. of *S*. *novaehollandiae*	1.21	3.04	1.51
Wt of *S*. *novaehollandiae*	0.42	3.14	0.99
Mean individual wt of *S*. *novaehollandiae*	2.51	0.10	0.09
No. of monacanthids	0.62	0.73	0.18
No. of *Pseudocaranx wrighti*	0.45	4.74*	0.74
No. of *Parapriacanthus elongatus*	0.31	1.26	0.60
No. of *Upeneichthys vlamingii*	0.05	0.11	2.74
No. of *Repomucenus calcaratus*	1.06	1.62	1.68
No. of *Parequula melbournensis*	1.33	0.06	2.13

^a^ U8 and U10 indicate under 8 or 10 *M*. *latisulcatus* per pound (454 g), respectively.

^b^ *Melicertus latisulcatus* industry categories are counts per pound.

*p < 0.05

**p < 0.01

***p < 0.001;–, insufficient data for analyses.

**Fig 6:**
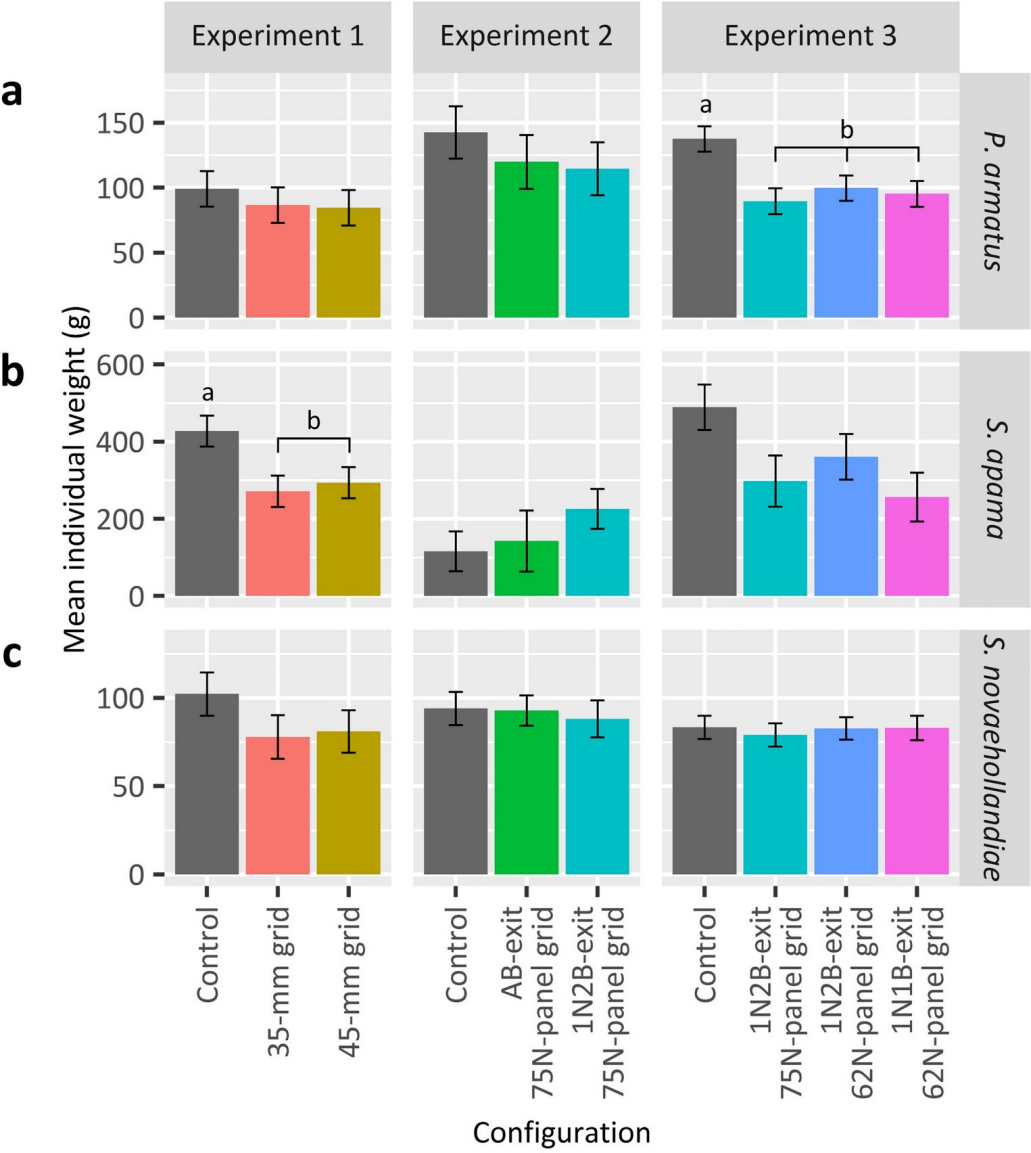
**Predicted mean individual weights (± SE) of (a) *Portunus armatus*, (b) *Sepia apama* and (c) *S*. *novaehollandiae* by configuration and experiment.** Where there was a significant configuration effect (*p* < 0.05) among a species within an experiment, letters above bars denote the false-discovery-rate (FDR) adjusted pairwise comparisons (*p* < 0.05).

False-discovery rate pairwise comparisons revealed that, compared to the control, trawls with the 35- and 45-mm grids similarly retained significantly lower numbers and weights of *P*. *armatus* (predicted means reduced by up to 67 and 56%, respectively) and elasmobranchs (up to 96 and 97%), weights of *S*. *apama* (73 and 52%) at smaller individual weights (428 ± 40 vs 272 ± 41 and 294 ± 40 g), and weights of *I*. *peronii* (88 and 80%), porifera (91 and 89%) and total bycatch (61 and 52%) (*p <* 0.05; Figs 5B–5G and 6A). The only variable not similarly affected was the number of *S*. *apama*, with a significant reduction (56%) by the 35-mm grid only (FDR, *p <* 0.05).

### Experiment 2: Assessing escape-exit area (control vs AB-exit 75N-panel vs 1N2B-exit 75N-panel grids)

To alleviate the risk of injury from falling debris during retrieval, the crew routinely checked the grid extensions and, as required, extracted debris and brushed the grid clean as the extension was hauled aloft. There were no deployment or on-board handling issues, and clearly less clogging around the frame line of the 1N2B-exit grid than that with the smaller AB-exit. No weed/debris accumulated near the base of the grid with the horizontal support at the top third.

As in experiment 1, LMMs failed to detect significant differences among configurations for the industry categories and total weights of *M*. *latisulcatus* (*p* > 0.05), except for those that were soft and broken, with a 44% lower predicted mean catch by the AB-exit 75N-panel grid than the control (FDR, *p* < 0.05; Fig 4B). Notwithstanding the above, there also was a trend of smaller total catches of *M*. *latisulcatus* with the 1N2B-exit 75N-panel grid than the control (by 14%; Fig 5A).

Significant configuration effects were detected for a similar set of bycatch species/groups to those identified in experiment 1, including the numbers and weights of *P*. *armatus* and elasmobranchs, and weights of porifera and total bycatch, but also the mean individual weight of *P*. *armatus* and number of *P*. *wrighti* (LMMs, *p* < 0.05; Table 2). No other bycatch species were affected by configuration (LMMs, *p* > 0.05; Table 2). Unlike in experiment 1, there were also no significant effects of configuration on the weights of *I*. *peronii* or the number, weight and mean individual weight of *S*. *apama* (LMMs, *p* > 0.05; Table 2). However, both species were retained in very low densities (i.e. <1 per deployment) across the trawled area.

In terms of the directions of significant differences, compared to the control, the AB- and 1N2B-exit 75N-panel grids caught significantly lower numbers and weights of *P*. *armatus* (by up to 67 and 63%, respectively) and elasmobranchs (up to 98% for both grids), and weights of porifera (91 and 93%) and total bycatch (54 and 62%) (FDRs, *p* < 0.05; Fig 5C and 5E–5G). The only variables not similarly affected were the number of *P*. *wrighti*, with significantly fewer (by 59%) caught by the 1N2B-exit 75N-panel grid than the AB-exit 75N-panel grid only (FDR, *p <* 0.05) and the mean individual weight of *P*. *armatus*, which could not be separated, although the 1N2B-exit 75N-panel grid caught smaller *P*. *armatus* (115 ± 20 g) than the AB-exit 75N-panel (120 ± 21 g) or control (143 ± 20 g) (FDR, *p* > 0.05; Fig 6A).

### Experiment 3: Assessing escape area and guiding panel length (control vs 1N2B-exit 75N-panel vs 1N2B-exit 62N-panel vs 1N1B-exit 62N-panel grids)

No technical issues were experienced with the three grids, except for one deployment needing to be repeated when the 1N2B-exit 72N-panel grid codend was empty (possibly owing to the trawl being twisted during deployment and not achieving bottom contact). There were also few issues with clogging among the grids, particularly for the 1N2B- and 1N1B-exit 62N-panel grids.

Similar to experiment 2, a significant configuration effect was detected for soft and broken *M*. *latisulcatus* (LMM, *p* < 0.01; Table 2), where lower weights were predicted with the 1N2B-exit and 1N1B-exit 62N-panel grids (34 and 33%, respectively) than the control (FDR, *p* < 0.05; Fig 4C). The only other *M*. *latisulcatus* category significantly affected by configuration was the U10 (LMM, *p* < 0.05; Table 2); however, this was not separated by the FDRs (*p* > 0.05; Fig 4C). By comparison, significant configuration effects were detected for the numbers, weights and mean individual weights of *P*. *armatus* and *S*. *apama*, number and weight of elasmobranchs, and weights of *I*. *peronii*, porifera and total bycatch (LMMs, *p* < 0.05; Table 2).

Compared to the control, the 1N2B-exit 75N-panel grid and the 1N2B- and 1N1B-exit 62N-panel grids caught significantly lower numbers and weights of *P*. *armatus* (by up to 78, 89 and 84%, respectively), *S*. *apama* (up to 90, 84 and 89%) and elasmobranchs (up to 100% for all grids), and weights of *I*. *peronii* (88, 96 and 96%), porifera (98% for all grids) and total bycatch (75, 77 and 79%) (FDRs, *p <* 0.05; Fig 5B–5G). The only significant difference between grids was for the number and weight of *P*. *armatus*, with the 1N2B-exit 62N-panel grid catching fewer than the 1N2B-exit 75N-panel grid (by up to 55%) (FDR, *p <* 0.05; Fig 5C).

The three grids similarly retained smaller *P*. *armatus* (90 ± 10, 100 ± 10 and 95 ± 10 g) than the control (138 ± 10 g) (FDR, *p* < 0.05; Fig 6A). While the 1N1B-exit 62N-panel grid also retained smaller *S*. *apama* (257 ± 64 g) than either the 1N2B-exit 75N-panel grid (298 ± 66 g), 1N2B-exit 62N-panel grid (361 ± 59 g) or control (489 ± 59 g), these differences were not significant (FDR, *p* > 0.05; Fig 6B). No other significant differences were detected (LMM, *p* > 0.05; Table 2).

## Discussion

The results from this study demonstrate the utility of cumulatively assessing technical changes to a generic Nordmøre-grid to approach a desired selectivity. The total bycatch reduction by the optimal design (~80%) was within the upper range of those observed for mechanical separators tested in other crustacean-trawl fisheries throughout the world (typically 50–90%) [5–8, 42, 43]. Further, the relative numbers of *P*. *armatus* and *S*. *apama* excluded by Kennelly and Broadhurst [7] were more than doubled in the present study from ~35 to >80%, with no loss of *M*. *latisulcatus*. These findings can be discussed in terms of species-specific morphologies and possible behavioural/mechanical responses to the various refinements.

### Grid evolution and possible escape mechanisms

Experiment 1 focused on identifying the optimal bar spacing between two grids: one with 35 mm, considered to be near the limit at which the largest *M*. *latisulcatus* (e.g. 39–49 mm carapace length with maximum carapace widths of 18–23 mm) [44] would be affected; and the other with 45 mm, tested by Kennelly and Broadhurst [7]. Neither grid significantly affected the catches of *M*. *latisulcatus* (although the 35-mm grid did retain slightly fewer in total), which largely can be attributed to the long, low-angled guiding panel directing the catch to the base of the similarly angled grid. This configuration provided minimal directional transition and clogging before sorting occurred across the entire surface of the grid [6–8]. Such a process clearly was sufficient for all *M*. *latisulcatus* to be more-or-less passively (considering their limited response to trawls) [45] orientated parallel to the bars and pass through. And this occurred despite a relatively (to many other penaeid fisheries) fast towing speed of ~1.9 m s^–1^.

Like *M*. *latisulcatus*, *S*. *apama* would have had considerable exposure to the grid surfaces and, considering their poor swimming speed, many also were probably orientated parallel to the bars. Consequently, their selection simply would have been a function of their mantle dimensions which, for experiment 1, meant only the 35-mm grid significantly reduced numbers. *Portunus armatus* exclusion would also be size-dependent, but their considerable morphological complexity means that spaces in the grids were likely to be less selective than for *M*. *latisulcatus* or *S*. *apama*.

In comparison, none of the teleosts caught were affected by the different bar spaces in experiment 1. Given that most teleosts were <14 cm TL and laterally compressed with widths considerably less than 35 mm, all simply passed through the grids. But, in terms of physical capabilities, many of these fish theoretically should have been able to avoid the grid if they had sufficient time to respond. Specifically, considering the horizontal distance of the grid (~1.7 m) and towing speed (~1.9 m s^–1^), organisms would need to swim a vertical distance of ~1.0 m at a speed of at least ~1.1 m s^–1^ to encounter the escape exit. Such swimming speeds should encompass the capabilities of most sizes caught [46]. Further, the proximity of the crab bag (~1 m) to the exit opening should have anteriorly displaced water (and caused a reduction in perceived flow), thereby assisting small teleosts to swim [47].

Considering the above, the lack of any behavioural escape by the teleosts implied insufficient visual cues and that bar spaces would have to be considerably narrower (or perhaps more visual) to affect their catches. Although not significant, narrower bars in the 35-mm grid promoted the escape of more *S*. *apama*, but the slight reduction in catches of *M*. *latisulcatus* implied concomitant losses with any further narrowing of spaces. For this reason, a bar spacing of ~38 mm was chosen as a compromise for the subsequent experiments (i.e. those that compared different escape exits and guiding-panel lengths).

Within the 38-mm grids, increasing the escape-exit opening also failed to affect teleost catches, although the predicted means for some of the abundant species varied and, in some cases, with greater numbers caught using the grid(s) than the control. Inspection of LMM residuals did not reveal any explanation for these inconsistent results; nonetheless, it was difficult to reconcile whether the larger-exit grids facilitated the escape of more teleosts.

Escape-exit area similarly had no apparent effects on *M*. *latisulcatus*, except for the soft and broken category, where lower weights were predicted with the AB- and 1N2B-exit 75N-panel grids (although the latter was not significant). Such a result presumably reflects fewer damaged *M*. *latisulcatus* due to the reductions of *P*. *armatus* in catches [11].

*Sepia apama* were encountered at low densities during experiment 2, precluding any assessment of their responses to the revised grids. Further, few insights were gained from the more consistently abundant *S*. *novaehollandiae* owing to the large inter-specific size difference. Therefore, the 1N2B-exit opening grid was reassessed in experiment 3, but with a further increase in the exit opening and reduction in the guiding panel length. The rationale for these changes was to increase the opportunities for anterior escape by reducing contact distance with the grid [4].

Consistent with the earlier experiments, configuration effects were detected in experiment 3 for the same bycatch species/groups (i.e. numbers and weights of *P*. *armatus*, *S*. *apama* and elasmobranchs, and weights of porifera and total bycatch). The actual refinements appeared to be particularly beneficial in that all three Nordmøre-grids yielded the largest reductions in these categories for the whole study, while also maintaining *M*. *latisulcatus* catches. With no one particular Nordmøre-grid consistently outperforming the others across all bycatch categories, the small variations manifested as slightly greater, but non-significant, reductions in total bycatch with each refinement.

An important consideration in interpreting the above observations is the potential for inter-experimental confounding of the sizes of organisms caught. Specifically, the mean individual weight of *S*. *apama* was larger in experiment 3 than in experiment 1; however, given that the difference was not significant, confounding effects might be small, particularly when compared to the disproportionately greater exclusion between configurations.

### Economic and ecological benefits of using Nordmøre-grids

The catch dynamics associated with the tested Nordmøre-grids also revealed that an economic benefit should accrue to the SGPF as a result of retaining fewer *P*. *armatus* in trawls and presumed reductions in the associated damage to *M*. *latisulcatus*. Densities of both species are temporally and spatially variable, making extrapolations difficult. But, if the reduction in the soft and broken category of *M*. *latisulcatus* was proportionally allocated among all sizes, then based on recent landings and prices [48] Nordmøre-grids would increase value of the catch (not accounting for other costs associated with constructing and maintaining the grids) to the fishery by some A$0.44M per year. Such revenue would more than offset any concomitant loss of *I*. *peronii* catches (~A$0.04M per year).

The net impact of using the Nordmøre-grids on the *P*. *armatus* stock is less clear. While reductions in the bycatch of any species might normally be assumed to benefit subsequent stock(s), there is an absence of data on the fate of *P*. *armatus* escaping grids and some evidence of increased productivity of this scavenger species with trawl intensity [22, 23, 49]—the latter presumably due to higher volumes of discards being available as food, including its conspecifics via cannibalism. Notwithstanding the uncertainty of how the *P*. *armatus* stock might be impacted, the reduction in the remaining bycatch (i.e. excluding *P*. *armatus*) using the Nordmøre-grids—estimated by deduction to be in the order of 70–80%—is likely to have some positive ecological impacts.

### Technical considerations and future research

Throughout the study, there were few issues (2% of replicate tows) with debris clogging the extensions or grids or repeated deployments due to twisting of the extension section. With more experience using grids, even fewer issues may arise. However, while a large grid appears to be a fundamental design component of a BRD for this fishery [7], the skipper noted this also made it cumbersome. Like many other fishers who are new to using such modifications, Spencer Gulf fishers are apprehensive of large mechanical-type separators due to operational and safety concerns. Acknowledging these concerns, a next phase in this work would be to undertake trials of the preferred grid system across broader spatio-temporal scales and with more fishers in the fishery. Also, refinements to the 62N-panel grid involving lighter materials, like polymers, may be worthwhile. By including operational data with the assessments of catches, it should be possible to objectively gauge any concerns fishers have with using the system and perhaps modify deployment and on-board handling procedures so that they are more acceptable to industry.

### Conclusions

Combined with the previous work of Kennelly and Broadhurst [7], the present study highlights an incremental approach to the development of a Nordmøre-grid, whereby technical refinements were made over a series of experiments to identify the best grid configuration for the SGPF with respect to maximising reductions of *P*. *armatus*, *S*. *apama* and total bycatch, while maintaining *M*. *latisulcatus* catches. The greatest reductions of *P*. *armatus*, *S*. *apama*, elasmobranchs, porifera and total bycatch (with no loss of *M*. *latisulcatus*) were achieved by a large, low-angled Nordmøre-grid with 38-mm bar spaces, a support bar two thirds up the length, a guiding panel terminating ~0.6 m anterior to the grid base, and a large escape exit. Further, probably due to fewer *P*. *armatus* being caught, damage to *M*. *latisulcatus* was reduced, resulting in a better quality and value of the retained target species. These results clearly demonstrate the potential for improved selectivity in this fishery using a Nordmøre-grid—primarily by mechanical exclusion of bycatch species from the target species largely owing to size and/or morphological differences.

## Supporting information

S1 Table Scientific and common names and numbers (*n*) of bycatch species caught during experiments 1, 2 and 3, and, for subsampled teleosts, median and range of total lengths. (DOCX)

S1 File Numbers and/or weights of targeted, by-product and bycatch species/species groups caught during experiments 1, 2 and 3, and geographic coordinates of trawl paths. For columns J–AK, numbers of organisms are indicated by column headers labelled with the prefix ‘num’; all other values are total weights in kg.
(XLSX)
