# Supplementary material for: Refining a Nordmøre-grid bycatch reduction device for the Spencer Gulf penaeid-trawl fishery
Source: PLoS One. 2018 Nov 21;13(11):e0207117. doi: 10.1371/journal.pone.0207117 (PMC6248960; doi:10.1371/journal.pone.0207117)
Supplement: S1 Table — (DOCX) [file pone.0207117.s001.docx]

**S1 Table. Scientific and common names and numbers (*n*) of bycatch species caught during experiments 1, 2 and 3, and, for subsampled teleosts, median and range of total lengths.**

| **Family (or next lowest taxonomic level)** | **Scientific name** | **Common name** | ***n*_1_** | ***n*_2_** | ***n*_3_** | **Total length (cm)** | |
| --- | --- | --- | --- | --- | --- | --- | --- |
|  |  |  |  |  |  | **Median** | **Range** |
| *Teleosts* |  |  |  |  |  |  |  |
| Apogonidae | *Vincentia badia* | Scarlet cardinalfish | 60 | 684 | 105 | 6.5 | 4.5–10.5 |
| Callionymidae | *Repomucenus calcaratus* | Spotted dragonet | 1425 | 4392 | 2177 | 12.5 | 8.5–21.5 |
| Carangidae | *Pseudocaranx wrighti* | Skipjack trevally | 1923 | 19057 | 15457 | 11.5 | 5.5–17.5 |
|  | *Trachurus declivis* | Common jack mackerel | - | 34 | 117 | 14.8 | 11.5–20.5 |
|  | *Trachus novaezelandiae* | Yellowtail scad | 61 | 21 | - | 16.0 | 13.0–17.5 |
| Chaetodontidae | *Chelmonops curiosus* | Western talma | - | - | 17 | 11.0 | 9.5–12.5 |
| Clupeidae | *Hyperlophus vittatus* | Sandy sprat | 36 | 53 | - | 8.5 | 7.0–10.5 |
|  | *Sardinops sagax* | Australian sardine | 68 | 141 | - | 12.0 | 6.5–14.5 |
| Cynoglossidae | *Cynoglossus broadhursti* | Southern tongue sole | 86 | 60 | 74 | 20.5 | 9.5–23.0 |
| Diodontidae | *Diodon nicthemerus* | Globefish | 2 | 8 | 4 | nr | nr |
| Engraulidae | *Engraulis australis* | Australian anchovy | 183 | 114 | 38 | 10.5 | 7.0–13.0 |
| Gempylidae | *Thyrsites atun* | Barracouta | - | 72 | - | 28.3 | 27.0–29.0 |
| Gerreidae | *Parequula melbournensis* | Silverbelly | 1048 | 3797 | 965 | 9.5 | 6.0–18.0 |
| Gobiesocidae | Not identified | Clingfish | - | 21 | - | 9.0 | 8.0–10.0 |
| Gonorynchidae | *Gonorynchus greyi* | Beaked salmon | 14 | 114 | 17 | 24.3 | 20.0–27.0 |
| Hemiraphidae | *Hyporhamphus melanochir* | Southern garfish | 26 | - | 29 | 14.3 | 10.0–14.5 |
| Monacanthidae | *Acanthaluteres spilomelanurus* | Bridled leatherjacket | 12519 | 21049 | 6213 | 10.0 | 3.0–21.0 |
|  | *Acanthaluteres vittiger* | Toothbrush leatherjacket |  |  |  |  |  |
|  | *Brachaluteres jacksonianus* | Southern pygmy leatherjacket |  |  |  |  |  |
|  | *Eubalichthys mosaicus* | Mosaic leatherjacket |  |  |  |  |  |
|  | *Scobinichthys granulatus* | Rough leatherjacket |  |  |  |  |  |
|  | *Thamnaconus degeni* | Bluefin leatherjacket |  |  |  |  |  |
| Mullidae | *Upeneichthys vlamingii* | Bluespotted goatfish | 2656 | 5362 | 1248 | 11.5 | 4.5–21.0 |
| Odacidae | *Neoodax balteatus* | Little weed whiting | 18 | 55 | 39 | 8.5 | 7.0–12.0 |
| Ostraciidae | *Aracana ornata* | Ornate cowfish | 40 | 31 | - | 10.3 | 10.0–11.0 |
|  | *Aracana aurita* | Shaw’s cowfish |  |  |  |  |  |
| Paralichthyidae | *Pseudorhombus jenynsii* | Smalltooth flounder | 532 | 251 | 608 | 18.8 | 14.0–37.0 |
| Pegasidae | *Pegasus lancifer* | Sculptured seamoth | - | 47 | - | 7.5 | 7.0–8.5 |
| Pempheridae | *Parapriacanthus elongatus* | Elongate bullseye | 6204 | 1884 | 1698 | 8.5 | 5.0–11.5 |

*Continued.*

| **Family (or next lowest taxonomic level)** | **Scientific name** | **Common name** | ***n*_1_** | ***n*_2_** | ***n*_3_** | **Total length (cm)** | |
| --- | --- | --- | --- | --- | --- | --- | --- |
|  |  |  |  |  |  | **Median** | **Range** |
| Pentacerotidae | *Parazanclistius hutchinsi* | Short boarfish | - | 42 | 1 | 11.5 | 10.5–12.5 |
| Pinguipedidae | *Parapercis haackei* | Wavy grubfish | 130 | 52 | 50 | 7.0 | 6.0–14.0 |
| Platycephalidae | *Platycephalus aurimaculatus* | Toothy flathead | 498 | 1122 | 389 | 22.8 | 9.5–35.0 |
|  | *Platycephalus richardsoni* | Tiger flathead |  |  |  |  |  |
|  | *Thysanophrys cirronasa* | Tasselsnout flathead |  |  |  |  |  |
| Sillaginidae | *Sillaginodes punctata* | King george whiting | 57 | 590 | 230 | 24.5 | 21.0–33.5 |
|  | *Sillago bassensis* | Southern school whiting | - | 173 | - | 18.0 | 17.0–22.5 |
| Sparidae | *Pagrus auratus* | Snapper | - | 0 | 20 | 9.5 | 9.5–9.5 |
| Sphyraenidae | *Sphyraena novaehollandiae* | Snook | - | 2 | - | nr | nr |
| Syngnathidae | *Leptoichthys fistularius* | Brushtail pipefish | - | 63 | - | 25.3 | 22.5–28.5 |
|  | *Stigmatopora nigra* | Spotted pipefish |  |  |  |  |  |
| Terapontidae | *Pelates octolineatus* | Western striped grunter | 323 | 100 | 463 | 16.0 | 6.0–20.0 |
| Tetraodontidae | *Polyspina piosae* | Orangebarred puffer | - | - | 52 | 7.5 | 6.5–8.5 |
|  | *Contusus brevicaudus* | Prickly toadfish | 12 | 111 | 2 | 17.3 | 12.0–22.5 |
|  | *Tetractenos glaber* | Smooth toadfish |  |  |  |  |  |
| Triglidae | *Lepidotrigla papilio* | Spiny gurnard | 572 | 1662 | 147 | 9.0 | 6.5–13.0 |
| Suborder Scorpaenoidei | *Gymnapistes marmoratus* | Soldier | 1235 | 1513 | 885 | 8.5 | 5.5–13.5 |
|  | *Maxillicosta scabriceps* | Little gurnard perch |  |  |  |  |  |
|  | *Neosebastes bougainvilii* | Gulf gurnard perch |  |  |  |  |  |
| *Chondrichthyans* |  |  |  |  |  |  |  |
| Callorhinchidae | *Callorhinchus milii* | Elephantfish | - | 1 | - | nr | nr |
| Dasyatidae | *Dasyatis thetidis* | Black stingray | - | 2 | - | nr | nr |
| Heterodontidae | *Heterodontus portusjacksoni* | Port Jackson shark | 31 | 61 | 20 | nr | nr |
| Hypnidae | *Hypnos monopterygium* | Coffin ray | 1 | - | - | nr | nr |
| Myliobatidae | *Myliobatis tenuicaudatus* | Southern eagle ray | - | 4 | - | nr | nr |
| Orectolobidae | *Orectolobus maculatus* | Ornate wobbegong | 14 | - | 3 | nr | nr |
| Rhinobatidae | *Aptychotrema vincentiana* | Western shovelnose ray | - | 1 | - | nr | nr |
|  | *Trygonorrhina dumerilii* | Southern fiddler ray | - | 3 | - | nr | nr |
| Squatinidae | *Squatina australis* | Australian angel shark | - | 2 | 2 | nr | nr |
| Urolophidae | *Urolophus paucimaculatus* | Sparsely spotted stingaree | 4 | - | - | nr | nr |
| Superorder Batoidea | Not identified | Rays and skates | 4 | 28 | 3 | nr | nr |

*Continued.*

| **Family (or next lowest taxonomic level)** | **Scientific name** | **Common name** | ***n*_1_** | ***n*_2_** | ***n*_3_** | **Total length (cm)** | |
| --- | --- | --- | --- | --- | --- | --- | --- |
|  |  |  |  |  |  | **Median** | **Range** |
| *Cephalopods* |  |  |  |  |  |  |  |
| Octopodidae | *Octopus kaurna* | Southern sand octopus | - | 17 | 20 | nr | nr |
| Ommastrephidae | *Nototodarus gouldi* | Gould's squid | - | 29 | - | nr | nr |
| Sepiadariidae | *Sepioloidea lineolata* | Striped pyjama squid | - | 12 | 6 | nr | nr |
| Sepiidae | *Sepia apama* | Giant Australian cuttlefish | 227 | 15 | 165 | nr | nr |
|  | *Sepia braggi* | Slender cuttlefish | - | 1 | - | nr | nr |
|  | *Sepia novaehollandiae* | New Holland cuttlefish | 407 | 126 | 265 | nr | nr |
| *Crustaceans* |  |  |  |  |  |  |  |
| Carcinidae | *Nectocarcinus integrifons* | Rough rock crab | - | 44 | - | nr | nr |
| Majidae | Not identified | Spider crab | - | 8 | - | nr | nr |
| Penaeidae | *Metapenaeopsis* sp. | Velvet shrimp | 889 | 2697 | 344 | nr | nr |
| Portunidae | *Ovalipes australiensis* | Common sand crab | - | 1240 | - | nr | nr |
|  | *Portunus armatus* | Blue swimmer crab | 13107 | 7192 | 4524 | nr | nr |
| Squillidae | *Erugosquilla grahami* | Mantis shrimp | 7 | 850 | 43 | nr | nr |
| *Bivalves* |  |  |  |  |  |  |  |
| Mytilidae^a^ | Not identified | Mussel | *29 kg* | *24 kg* | *89 kg* | nr | nr |
| Pectinidae | Not identified | Scallop | 15 | 12 | 13 | nr | nr |
| Pinnidae | *Pinna bicolor* | Razor clam | - | - | 3 | nr | nr |
| Order Veneroida | Not identified | Cockle | 16 | 16 | - | nr | nr |
| *Echinoderms* |  |  |  |  |  |  |  |
| Holothuriidae | *Holothuria hartmeyeri* | Handsome sea cucumber | 1 | 5 | - | nr | nr |
| Class Asteroidea | Not identified | Starfish | - | 2 | 2 | nr | nr |
| Class Echinoidea | Not identified | Sea urchin | - | 16 | 5 | nr | nr |

^a^ Mytilidae were not counted, only weighed (weights are shown in italic).

-, not caught; nr, not recorded.
